# Revisiting unstable disability and the fluctuations of frailty: a measurement burst approach

**DOI:** 10.1093/ageing/afae170

**Published:** 2024-08-08

**Authors:** Erwin Stolz, Anna Schultz, Hannes Mayerl, Regina Roller-Wirnsberger, Andrew Clegg

**Affiliations:** Institute of Social Medicine and Epidemiology, Medical University of Graz, Graz, Austria; Institute of Social Medicine and Epidemiology, Medical University of Graz, Graz, Austria; Institute of Social Medicine and Epidemiology, Medical University of Graz, Graz, Austria; Department of Internal Medicine, Medical University of Graz, Graz, Austria; Academic Unit for Ageing and Stroke Research, Bradford Institute for Health Research, University of Leeds, Leeds LS2 9JT, UK

**Keywords:** disability, frailty, short-term fluctuations, intra-individual variability, measurement burst design, older people

## Abstract

**Background:**

It has been hypothesised that frailty is the root cause of clinically observed but rarely systematically measured unstable disability among older adults. In this study, we measure the extent of short-term disability fluctuations and estimate their association with frailty using intensive longitudinal data.

**Methods:**

Repeated measurements of disability were collected under a measurement burst design in the FRequent health Assessment In Later life (FRAIL70+) study. A total of 426 community-dwelling older adults (70+) in Austria were interviewed about difficulties with basic, instrumental and mobility-related activities of daily living biweekly up to a total of 14 times in two measurement bursts (2891 and 2192 observations). Baseline frailty was assessed with both physical frailty (FP) and the frailty index (FI). Disability fluctuations were measured with the intra-individual interquartile range (iIQR) and estimated with a two-step generalised mixed regression procedure.

**Results:**

Fewer participants were frail at baseline according to FP (11%) than FI (32%). Frail study participants reported not only more severe disability but also had more short-term disability fluctuations (iIQR = 1.0–1.5) compared with their robust counterparts (iIQR = 0). Regression models indicated that baseline frailty was associated with 2–3 times larger short-term disability fluctuations, which were also more prevalent among women, and increased with age and disability severity.

**Conclusion:**

Compared with those who were robust, frail older adults were characterised by not only more severe but also more unstable disability. Short-term disability fluctuations are closely tied to disability severity. Future studies should assess both stressors that may cause disability fluctuations among frail older adults as well as their potential consequences to inform frailty-centred care.

## Key Points

- Frailty is associated with increasingly unstable disability over the course of weeks.
- On average, disability fluctuated by one activity difficulty among older adults with frailty.
- Disability fluctuations were also more prevalent among women and increased with age and disability severity.

## Introduction

### Background

Frailty results from a cumulative decline in multiple physiological systems, and is defined as a state of increased vulnerability to (minor) stressors resulting in adverse health outcomes among older adults [1]. Frailty increases with age [2], and is fairly common among older adults: a meta-analysis estimated that depending on the instrument, 12%–24% of community-dwelling older adults (50+) are frail [3]. Frailty has major implications for clinical practice and public health [2], and can help in identifying older individuals at risk for falls [4], disability [5] and mortality [6], which in turn can guide targeted care efforts [7].

Research into frailty has proliferated during last two decades fuelled by two seminal papers [8, 9], which outlined, what would later become the two dominant operationalisations of frailty [10], i.e. physical or phenotype frailty (FP) and the frailty index (FI) [11]. FP [8, 12–14] is defined as a clinical syndrome of having three or more out of five designated criteria (weight loss, exhaustion, poor grip strength, slow gait speed and low physical activity). The FI [9, 15–17], on the other hand, measures the degree of frailty in a continuous manner by summing up a large number (30+) of non-predetermined, age-related health deficits, which can subsequently also be used to classify frail and robust older adults [18].

The cumulative decline across multiple physiological systems that characterises frailty can potentially manifest as unstable disability [19]. This can be understood as short-term fluctuations in functioning when small precipitants such as a change in the weather, the drug regime or an infection results in a sudden increase in disability—typically loss of independence in one or more activities of daily living—that then reverses back over the following days and weeks. Although such disability fluctuations (I) have been reported by medical practitioners, (II) have been integrated into conceptualisations of frailty [1], and (III) might have negative consequences for both disabled older adults and familial caregivers as well as professional care providers—e.g. with regard to assessment of disability and predictability of everyday life—the hypothesised association between frailty and unstable disability has rarely been assessed empirically. This is perhaps surprising, as (physical) frailty has been conceptualised and empirically confirmed as an important cause of disability [5, 8, 12] among older adults, and late-life disability has been shown to be dynamic [20–23]. One reason why these two strands of research have rarely been connected is likely because the intensive longitudinal data required to assess instability or fluctuations, i.e. multiple repeated measurements of disability over a short-period of time, are generally scarce.

### Objectives

The main objective of this study was to address this evidence gap by assessing whether frailty is associated with short-term fluctuations in disability among older adults using newly available intensive longitudinal data from a nationwide measurement burst study.

## Methods

### Study design

In the FRequent health Assessment In Later life (FRAIL70+) study, longitudinal data were collected under a measurement burst design [24, 25], where multiple bursts consisting of repeated intensive longitudinal assessments allow for the assessment of both short-term fluctuations (within bursts) and long-term changes (between bursts).

### Setting and participants

A professional survey agency collected health-related information in a nation-wide sample of 426 community-dwelling older adults aged 70 years and above in Austria (Supplementary Methods 1). Two measurement bursts (burst 1: *n* = 426, burst 2: *n* = 378), each running about 3 months (mean run time: burst 1 = 87 ± 13 days; burst 2 = 76 ± 11 days), were conducted 12 months apart between September of 2021 and January 2023 (Supplementary Figure 1). In each burst, participants were interviewed approximately every 2 weeks, up to seven times. The first interview of each burst was conducted in person in the older adult’s home; follow-up interviews were generally conducted by phone, except for a 10% subsample for which all interviews during the first burst were in-person in order to assess potential interview mode effects. Before participation, interviewers described content, length and required information of the study, ensured anonymity of all personal data and obtained written consent. This study was approved by the Ethics Committee of the Medical University of Graz (EK-number: 33-243 ex 20/21 1035-2021).

### Variables

Disability was measured repeatedly based on difficulties (no/yes) participants reported having had during the last 2 weeks in six activities of daily living [26] (ADLs: dressing, walking across room, bathing/showering, eating, get in/out of bed, use toilet), four instrumental activities of daily living [27] (IADLs: prepare a warm meal, shop groceries, use telephone, take medication) and four mobility-related activities [28] (walking 100 m, climb one flight of stairs, extend arms above should level, lift/carry 5 kg) (Supplementary Tables 1 and 2). The overall level of disability at each measurement point was calculated as the sum of present activity difficulties (range = 0–14) [29, 30]. Internal consistency reliability of this sum index based on confirmatory factor analysis was adequate as estimated by coefficient omega (0.75 in both bursts). Test–retest reliability was also adequate as indicated by the average Spearman correlation coefficient between adjacent measurements of 0.78 (burst 1) and 0.83 (burst 2). For more details, see Supplementary Methods 2.

Frailty was measured in the first interview of the first measurement burst by both (i) FP [8] and (ii) the FI [16]. (i) Calculation of FP was based on five criteria: slow walking speed, muscle weakness, low physical activity, exhaustion and low appetite or being underweight as proxy variables [31] for weight loss. Slow walking speed was measured as belonging to the lowest 20% in gait speed (minimum of two trials over 2.5 m) in seconds. Muscle weakness was based on previously specified sex- and body mass index (BMI)-specific grip strength values (Supplementary Table 3), measured as the maximum reading (in kg) over four trials (two per each hand) with a handheld dynamometer (Smedley S Dynamometer, TTM, Tokyo, 100 kg). Participants who could not perform the walking speed or grip strength test were considered to fulfil the respective criterion. Low physical activity was based on an item asking the participants how often they engaged in activities that require a low or moderate level of energy such as gardening, cleaning the car or doing a walk. If participants answered less often than weekly, they were considered exhibiting low physical activity. The exhaustion criterion was based on two items from the Center for Epidemiologic Studies Depression Scale (CES-D). The criterion was met if participants answered ‘often or always’ when asked how often in the last 2 weeks they felt ‘Everything I did was an effort’ or ‘I could not get going’. Finally, low appetite/being underweight was based on reporting often or always to have poor appetite or a BMI < 18.5. Participants were classified as frail if they met three or more of these criteria. (ii) Calculation of the FI was based on 33 health deficits (Supplementary Table 3), which included self-reported information on chronic diseases and polypharmacy, somatic symptoms such as pain, tiredness and dizziness, bedrest and falls, depressive symptoms (CES-D), sensory impairment, physical inactivity, poor self-rated health as well as poor physical (slow gait speed, slow chair rise test scores, weak grip strength), and cognitive performance (attention, short-term memory). FI calculation followed standard protocol [32, 33] except that no disability measures were included in the FI. The FI was calculated for all participants by dividing the sum of the health deficit score by the total number of health deficits measured, e.g. 8/35 = 0.23. As cut-off value to differentiate between non-frail and frail older adults, 0.25 [18] was used.

Sociodemographic variables included sex (male/female), chronological age (years), level of completed education (low = compulsory education, medium = vocational training, high = high school or higher) and living alone (no/yes).

### Statistical methods

Given the discreteness and skew of the outcome (i.e. disability), we calculated the intra-individual median (iMD) to describe the average disability severity and the intra-individual interquartile range (iIQR) to quantify short-term disability fluctuations for each burst. Specifically, the iIQR quantifies the middle 50% of biweekly within-person disability variability. Then, we assessed differences in iMD and iIQR by baseline FP/FI status. To isolate the effect of baseline frailty on short-term disability fluctuations from sociodemographic characteristics, particularly chronological age, we used a two-step approach: first, given the discreteness, skew, potential floor effects and upper bound (=14) of the outcome, we tested several generalised linear mixed model variants to analyse disability severity, of which the zero-inflated beta-binomial model clearly provided the best fit (Supplementary Table 4). Then, we extracted observation-level residuals from these models, which represent the vertical deviations from individual-specific (random intercept and slope) disability trajectories within each measurement burst (Supplementary Figure 2). The absolutized observation-level residuals were then modelled as outcomes in a second step using again mixed regression models. For more details on the statistical approach, see Supplementary Methods 3. Finally, there were some missing values in a few participants (*n* = 12, 2.8%) in one or more of the five FP criteria, i.e. when participants did not provide a valid answer or did not want to perform a test, which we addressed with a random forest imputation procedure, which classified one additional participant as frail and the remaining 11 as robust (out-of-bag error = 0.04).

## Results

### Participants and descriptive data

From the 426 participants at baseline, 64.6% were women, 66% lived alone and mean age was 77.3 (SD = 5.4, range = 70–96) years. Low education was reported by 19.3%, medium by 54.2% and high by 26.5%. In the first measurement burst, 425 participants provided 2891 repeated observations of disability, and 375 participants (88% of the baseline sample) provided another 2192 observations in the second burst. Fewer older adults were classified as frail according to FP (11.0%, *n* = 47) than according to the FI instrument (34.5%, *n* = 147). Overlap between the two frailty approaches was moderate (Cohen’s kappa = 0.36): 277 individuals were classified as robust by both instruments, 45 older adults were frail according to both instruments, 102 were frail only according to the FI and 2 only according to FP. More information on sample characteristics is provided in Table 1.

**Table 1:** Sample characteristics (baseline)

	Total	Physical frailty		Frailty index	
		Robust	Frail	Robust	Frail
	*N* (%)	*N* (%)	*N* (%)	*N* (%)	*N* (%)
Men	151 (35.4)	139 (92.1)	12 (7.9)	113 (74.8)	38 (25.2)
Women	275 (64.6)	240 (87.3)	35 (12.7)	166 (60.4)	109 (39.6)
Low education	82 (19.3)	67 (81.7)	15 (18.3)	33 (40.2)	49 (59.8)
Medium education	231 (54.2)	203 (87.9)	28 (12.1)	151 (65.4)	80 (34.6)
High education	113 (26.5)	109 (96.5)	4 (3.5)	95 (84.1)	18 (15.9)
Living alone	145 (34.0)	132 (91.0)	13 (9.0)	109 (75.2)	36 (24.8)
Living with others	281 (66.0)	247 (87.9)	34 (12.1)	170 (60.5)	111 (39.5)
	Median (IQR)	Median (IQR)	Median (IQR)	Median (IQR)	Median (IQR)
Age	76.8 (8.0)	76.3 (7.8)	79.9 (6.7)	75.3 (7.3)	78.8 (8.0)
Baseline disability	0 (2)	0 (1)	6 (4.5)	0 (1)	3 (4)

Unweighted data, *n* = 426. IQR = interquartile range.

### Main results

The distribution of the number of reported functional limitations was highly right-skewed in both bursts (Supplementary Figure 3), i.e. more than half of the community-dwelling participants reported no functional limitations, and, for those who reported any, most had difficulties with ≤4 activities. The proportion of participants who reported difficulties in any activity ranged between 37% and 44% in the first and 41% and 44% in the second burst. Consequently, the average iMD in the total sample was zero in both bursts. iMDs correlated strongly across bursts (*r* = 0.82), i.e. changes in iMD over 1 year were limited: among those who participated in both bursts, about two-thirds (64.0%) had no change, iMD decreased in 11.7% and increased in 24.3% of the participants. Median iIQR was 0 in the total sample and correlated substantially with iMD: *r* = 0.57 in burst 1 and 0.62 in burst 2.

**Figure 1:**
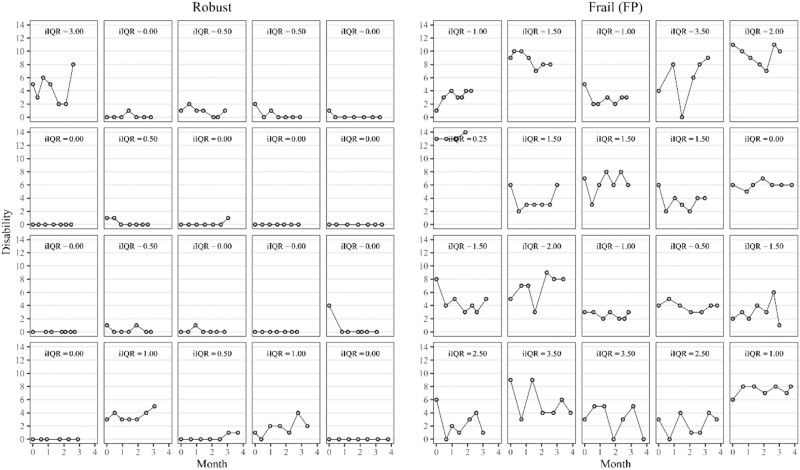
Repeatedly measured number of limitations for randomly selected robust and FP older adults during the first measurement burst. Each facet contains repeated disability measurements (points) of one individual older adult. FP = physical frailty, which describes average disability fluctuations (=middle 50% of intraindividual variability).

Both iMD and iIQR varied according to frailty status. Figure 1 illustrates the nature of disability fluctuations (iIQR), showing the measured number of difficulties during the first measurement burst for 40 randomly sampled participants, 20 of whom were robust and 20 were frail (FP). It shows that frail older adults were not only more severely disabled on average, but that they also had often highly stochastic (random) disability trajectories, sometimes including large increases and decreases across few weeks. These results extend to the full sample: frail older adults were not only more disabled according to both instruments (FP: iMD = 4 vs. 0; FI: iMD = 3 vs. 0) but also had more disability fluctuations, i.e. a median iIQR of 1–1.5 compared with 0 among robust older adults.

**Figure 2:**
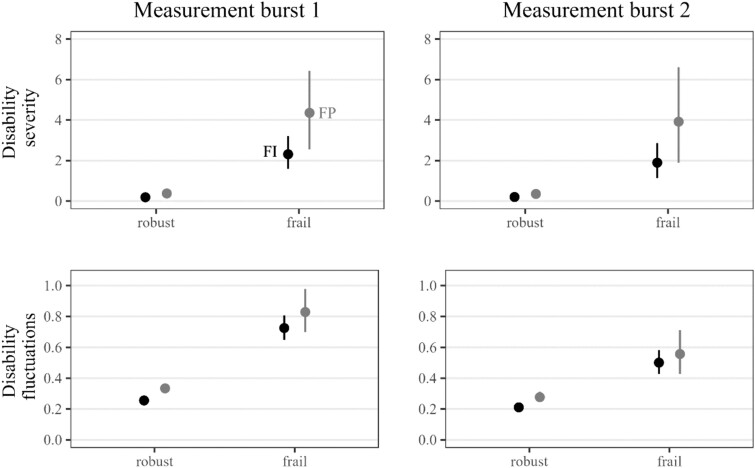
Estimated disability level and disability fluctuations by frailty status and measurement burst. Results based on regression models, point estimates represent the mean of the posterior distribution, and error bars represent 95% credible intervals. Estimates refer to women of average age with medium level education who live alone and with average disability severity. FP = phenotype frailty.

Figure 2 shows the predicted and adjusted effect of baseline frailty on disability severity and fluctuations in both bursts based on the regression models. While adjustment reduced differences attributable to frailty, there is still a clear difference: frail older adults were more severely disabled during both the first and the second burst and also had 2–3 times more short-term disability fluctuations. Results from the regression models (Supplementary Tables 4 and 5) further indicated that independently of frailty status, fluctuations were higher among women (+10%–57%) than men and increased with chronological age (+3%–6% per year) and particularly with disability severity (+47%–73% per activity difficulty).

## Discussion

### Key results

In this analysis, we have identified that frailty is associated with increasingly unstable disability over the course of weeks, along with more severe disability in general. Our findings were consistent across the FP and FI as the two established frailty models. Alongside frailty, older age, female sex and more severe disability were also associated with increasing disability fluctuations. We found that disability fluctuated by one activity difficulty among older adults with frailty, which is considerable against the average of 3–4 difficulties reported in this group. Strengths of the study include the measurement burst design including up to 14 repeated biweekly measurements of disability in a nation-wide sample of community-dwelling older adults and the dual operationalization of frailty.

In our study, disability severity, i.e. the number of difficulties reported, and the extent of short-term disability fluctuations were closely correlated, which, together with the increase with age of the latter, suggests that short-term disability fluctuations could be an inherent characteristic in the disabling process [34, 35]. It could be helpful clinically to view a single disability assessment in an older individual not as a definitive and stable indicator, but just one data-point in a person’s long-term disability trajectory, which becomes more unstable as disability severity increases [36].

### Limitations

Although the data stemmed from a nation-wide sampling frame and the response rate were comparable to the Survey of Health, Ageing and Retirement in Europe (SHARE) in Austria, there was evidence of sample selection. Hence, we expect that the prevalence of frailty is somewhat higher, and the level of disability as well as the extent of short-term disability fluctuations are also likely higher in the population than in the sampled older adults analysed here. It might be argued that (some of) the short-term fluctuations of disability we found in this study may be due to measurement error given that disability was self-reported. However, both internal consistency and test–retest reliability of overall disability were adequate in the current study, so that a majority of the biweekly disability changes over the course of the two 3-month periods likely represent true changes in disability. Moreover, both size and systematic patterning of short-term disability fluctuations suggest that they do not just reflect random measurement error. Nonetheless, future studies could rely on repeated performance measures to quantify short-term fluctuations in physical function over days and weeks [37] more precisely, or utilise newly available high-frequency measurements from wearables [38] to obtain insights into fluctuations in daily living among older adults.

While our results support that there is a relationship between frailty and unstable disability, it is unclear whether short-term disability fluctuations qualify as an adverse outcome in their own right, e.g. whether they impact well-being negatively beyond disability severity (e.g. due to reduced predictability of activities), and/or whether they have added prognostic value, e.g. with regard to nursing home admission or mortality. Also, disability may fluctuate even more frequently among older people with frailty, e.g. on a daily basis, which we could not capture with the biweekly assessments.

### Findings in context of previous research

Previous research [5, 8, 12] has tended to study the relationship between frailty and disability over extended time horizons between each assessment. One exemption to this is the precipitating events project (PEP) study [39], where disability has been measured monthly over many years among 754 health insurance plan members in the USA. Our results here are in line with an earlier PEP-based study [36], which also estimated disability fluctuations amounting to one ADL/IADL during the last 5 years of life. Before that, Hardy *et al*. [40] had examined disability in PEP to find that transitions between independence and ADL-disability were not only common, but that both the number of transitions between independence and disability as well as disability episodes (defined as independence followed by disability followed by recovery) were more prevalent among physically frail older adults compared with their robust counterparts. Our results are compatible with these findings, despite considerable differences in research design and data analysis, i.e. we used a continuous disability measurement, included both major operationalisations of frailty (FP [8] and FI [9]), and relied on a shorter but more intensive measurement burst design [24, 25] instead. In another PEP-based study, Gill *et al.* [41] delineated different disability subtypes, finding that FP predicted ‘long-term’ (6+ months), but also ‘recurrent’ (2 episodes, none lasting 6 months) and ‘unstable’ (3+ episodes, none lasting 6 months) disability, which again is compatible with our results.

### Interpretation

These novel findings lend support to the theoretical construct of frailty, providing new evidence for disability fluctuations that have been hypothesised to exist as a core feature. Notably, our data suggest that, in frailty, disability fluctuates over multiple weeks rather than just across 6 months or more as the usual time horizon of previous studies, and that these fluctuations are ongoing and stochastic.

### Generalisability

We expect our findings, based on a nationwide cohort, to apply to community-dwelling older adults, particularly those aged 70 years and over and those living in European countries similar to Austria.

## Supplementary Material

aa-24-0461-File002_afae170
