# Correction to: Revisiting unstable disability and the fluctuations of frailty: a measurement burst approach

**DOI:** 10.1093/ageing/afae203

**Published:** 2024-09-05

This is a correction to: Erwin Stolz, Anna Schultz, Hannes Mayerl, Regina Roller-Wirnsberger, Andrew Clegg, Revisiting unstable disability and the fluctuations of frailty: a measurement burst approach, *Age and Ageing*, Volume 53, Issue 8, August 2024, afae170, https://doi.org/10.1093/ageing/afae170.

In the originally published version of this article, author Andrew Clegg's first and last names were reversed. This error has been corrected.

