# Supplementary material for: Revisiting unstable disability and the fluctuations of frailty: a measurement burst approach
Source: Age Ageing. 2024 Aug 8;53(8):afae170. doi: 10.1093/ageing/afae170 (PMC11306319; doi:10.1093/ageing/afae170)
Supplement: aa-24-0461-File002_afae170 [file aa-24-0461-file002_afae170.docx]

Revisiting unstable disability and the fluctuations of frailty:

A measurement burst approach

**Supplementary Material**

Supplementary Methods 1: Sample selection and attrition (FRAIL70+)

Supplementary Figure 1: Disability assessment schedule

Supplementary Table 1: Prevalence of difficulty (in %) in activities (1^st^ burst)

Supplementary Table 2: Prevalence of difficulty (in %) in activities (2^nd^ burst)

Supplementary Methods 2: Reliability of disability instrument

Supplementary Table 3: Health deficits of the FI

Supplementary Table 4: Generalized linear mixed model comparison

Supplementary Figure 2: Illustration of disability fluctuations as observation-level residuals

Supplementary Methods 3: Statistical Analysis

Supplementary Table 5: Predictors of disability severity (µ) and fluctuations (σ) in the first burst

Supplementary Table 6: Predictors of disability severity (µ) and fluctuations (σ) in the second burst

Supplementary Figure 3: Distribution of the number of activity difficulties

Supplementary References

Supplementary Methods 1: Sample selection and attrition (FRAIL70+)

In total, 971 older adults were contacted based on previous participation in population-representative studies, of which 426 individuals agreed to participate (response rate=44%). When selected older adults who were successfully contacted did not want to participate in the study, they were asked to provide core demographic and health-related information (total of 4 items) in order to assess selectiveness of the sample. In comparison to participants, those who could or would not participate were more likely men (42.8% vs. 35.4%; χ²=5.3, df=1, p=0.021), had only minimum compulsory schooling (28.1% vs. 19.2%; χ²=20.3, df=2, p<0.001), and had poorer self-reported health (moderate = 37.5% vs. 28.4%, poor = 11.1% vs. 10.8%; χ²=23.9, df=4, p<0.001), but were of similar age (mean=77.4 vs. 77.3 years; F-statistic=0.19, df=1, p=0.663).

From the 426 participants at baseline (first interview in first measurement burst), 378 (88.7%) returned for the first interview in the second measurement burst. There were differences between participants who returned and those who dropped out between bursts with regard to socio-demographic characteristics, but these were not statistically significant (at p<0.05.) However, older adults who were frail at baseline according to either FP or FI were more likely not to return one year later: FP= 29.8% vs. 9.0%, Chi²=18.1, df=1, p<0.001; FI = 17.8% vs. 7.5%, Chi²=10.3, df=1, p=0.001). Also, older adults who dropped out were more severely disabled at baseline (difference = 1.7, F=20.9, df=408, p<0.001) and throughout the first burst (iMD difference = 1.1, F=11, df=401, p=0.001).

In conclusion, the FRAIL70+ sample is somewhat selective compared to the Austrian reference population 70+ (younger, more women, more higher-educated individuals, better health status) at baseline, and sample attrition between measurement bursts was in part health-related, rendering the second measurement burst more selective than the first.

Supplementary Figure 1: Disability assessment schedule


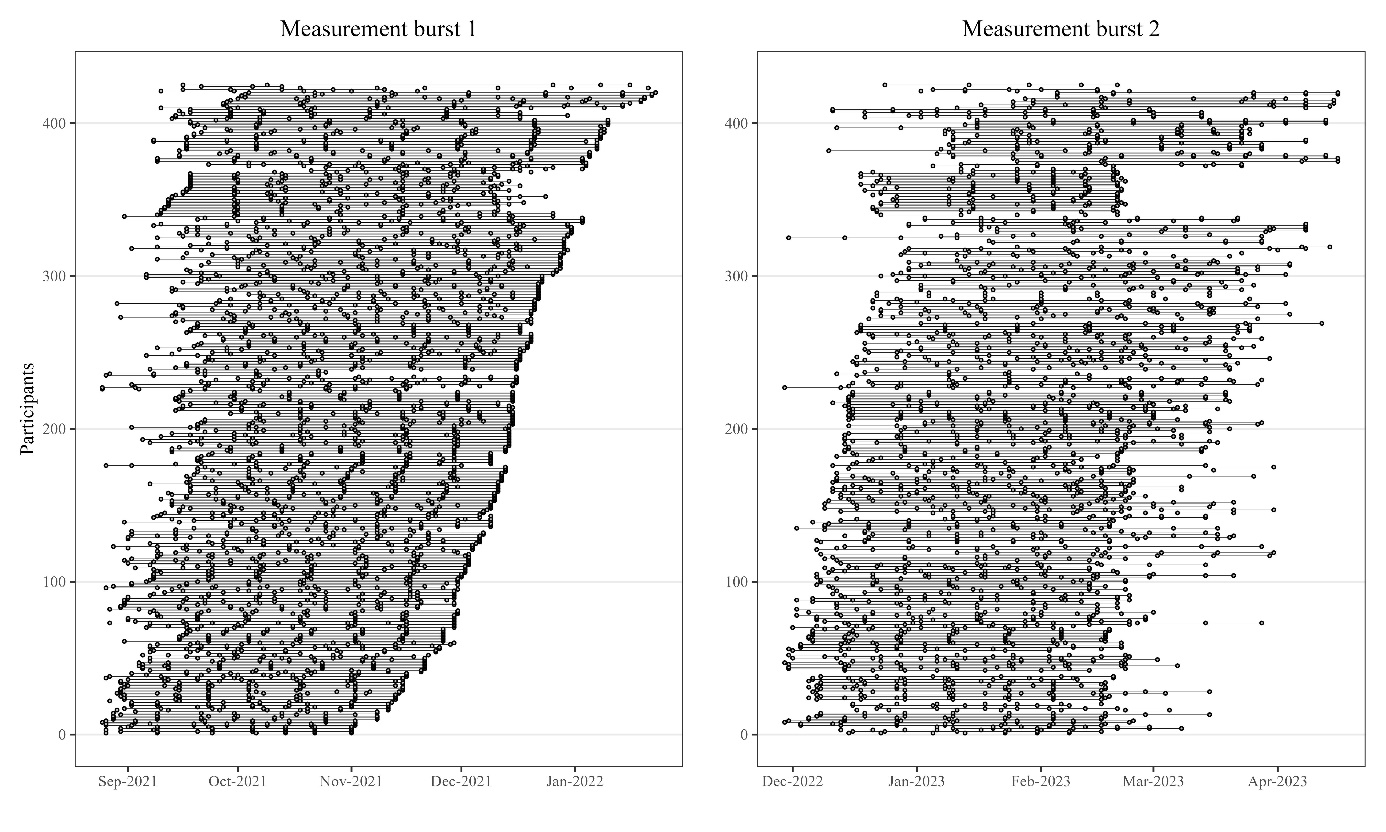


Each grey line shows a participant, each circle a completed interview.

Supplementary Table 1: Prevalence of difficulty (in %) in activities (1^st^ burst)

|  | Wave | | | | | | |
| --- | --- | --- | --- | --- | --- | --- | --- |
|  | 1 | 2 | 3 | 4 | 5 | 6 | 7 |
|  | n=425 | n=419 | n=419 | n=410 | n=406 | n=407 | n=406 |
|  |  |  |  |  |  |  |  |
| Dress, including shoes and socks | 12.0 | 8.8 | 11.0 | 11.7 | 7.9 | 11.3 | 11.6 |
| Walk across room | 7.8 | 5.0 | 5.0 | 4.2 | 2.5 | 4.0 | 4.7 |
| Bathing/showering | 9.6 | 8.4 | 8.4 | 8.5 | 5.4 | 7.4 | 7.1 |
| Eating (e.g., cutting meat or bread) | 3.8 | 3.3 | 3.6 | 2.4 | 2.2 | 2.0 | 3.0 |
| Get into or out of bed | 7.3 | 6.2 | 6.4 | 4.4 | 3.9 | 2.9 | 4.9 |
| Use toilet | 3.3 | 2.6 | 2.9 | 2.4 | 2.2 | 1.5 | 3.0 |
| Prepare warm meal | 5.2 | 5.0 | 4.3 | 5.6 | 4.5 | 5.0 | 7.2 |
| Shop for groceries | 11.6 | 11.2 | 12.7 | 11.0 | 10.7 | 11.9 | 12.9 |
| Using telephone | 1.6 | 1.7 | 2.4 | 2.0 | 1.0 | 0.5 | 0.7 |
| Take medication | 1.7 | 2.2 | 1.4 | 1.2 | 1.0 | 1.5 | 1.0 |
| Walk 100 meters | 12.3 | 13.4 | 13.5 | 17.7 | 11.9 | 12.8 | 13.4 |
| Climb one flight of stairs | 23.5 | 22.1 | 22.8 | 25.5 | 22.8 | 23.0 | 25.7 |
| Raise arms above shoulder level | 14.6 | 13.6 | 14.4 | 14.6 | 14.8 | 18.9 | 17.5 |
| Carry 5 kg or more | 27.7 | 27.6 | 26.6 | 28.2 | 29.1 | 30.1 | 30.8 |

Supplementary Table 2: Prevalence of difficulty (in %) in activities (2^nd^ burst)

|  | Wave | | | | | | |
| --- | --- | --- | --- | --- | --- | --- | --- |
|  | 1 | 2 | 3 | 4 | 5 | 6 | 7 |
|  | n=376 | n=347 | n=351 | n=323 | n=315 | n=286 | n=196 |
|  |  |  |  |  |  |  |  |
| Dress, including shoes and socks | 14.1 | 11.8 | 12.0 | 12.7 | 14.0 | 14.7 | 9.2 |
| Walk across room | 7.2 | 3.8 | 6.3 | 5.3 | 6.1 | 6.3 | 3.1 |
| Bathing/showering | 10.6 | 7.5 | 6.8 | 7.1 | 8.0 | 7.7 | 7.6 |
| Eating (e.g., cutting meat or bread) | 4.5 | 3.7 | 2.6 | 3.1 | 3.5 | 3.8 | 4.1 |
| Get into or out of bed | 7.4 | 4.6 | 4.5 | 4.0 | 4.1 | 4.2 | 3.0 |
| Use toilet | 3.7 | 3.5 | 0.9 | 1.2 | 2.5 | 3.1 | 2.0 |
| Prepare warm meal | 5.9 | 6.6 | 6.6 | 4.4 | 7.1 | 6.8 | 6.7 |
| Shop for groceries | 15.5 | 13.0 | 11.7 | 12.5 | 12.1 | 12.3 | 13.8 |
| Using telephone | 2.7 | 1.7 | 1.1 | 1.5 | 0.3 | 1.4 | 1.5 |
| Take medication | 3.2 | 2.9 | 2.0 | 1.9 | 2.2 | 2.5 | 2.6 |
| Walk 100 meters | 13.6 | 15.1 | 14.1 | 15.3 | 15.7 | 15.9 | 17.5 |
| Climb one flight of stairs | 24.5 | 25.8 | 24.4 | 24.9 | 26.0 | 29.7 | 27.7 |
| Raise arms above shoulder level | 16.7 | 15.6 | 16.8 | 16.1 | 17.8 | 21.3 | 14.7 |
| Carry 5 kg or more | 26.0 | 23.7 | 31.4 | 32.3 | 29.5 | 33.1 | 24.9 |

Supplementary Methods 2: Reliability of disability instrument

Internal consistency reliability was measured with coefficient omega^42^ based on confirmatory factor analysis (CFA). For both measurement bursts, separate CFA models were estimated, and factor loadings of activity difficulties were constrained to be equal across measurement occasions within each burst. Information from functional limitations were treated as categorical (0/1) and estimation was based on a diagonally weighted least squares procedure (WLSMV) with robust standard errors and listwise deletion. CFA was calculated with R-package lavaan (0.6-15)^43^, and internal consistency was calculated using R-package semTools (0.5-6). Robust fit indices indicated adequate model fit (and hence, construct validity) in both the first (Scaled Chi²-test=674, df=444, p<0.001, Robust Comparative Fit Index (CFI)=0.949, Robust Tucker-Lewis Index (TLI)=0.947, Robust Root Mean Square Error of Approximation (RMSEA)=0.050, Standardized Root Mean Square Residual (SRMR)=0.123) and the second measurement burst (Scaled Chi²-test=908, df=617, p<0.001, CFI=0.917, TLI=0.14, Root Mean RMSEA=0.062, SRMR=0.150. Based on CFA, we estimated an average coefficient omega across measurement occasions of 0.75 (in both bursts), which can be considered adequate.

Test-retest reliability of the disability sum index was based on Spearman correlation coefficients between adjacent measurement occasions within both measurement bursts, which amounted to 0.78 in the first, and 0.83 for the second burst, which again can be considered adequate.

Supplementary Table 3: Health deficits of the FI

| Health deficit | Coding | Prevalence at baseline in % | Missing data at baseline in % |
| --- | --- | --- | --- |
| Self-rated health | Excellent = 0, very good = 0.25, good = 0.50, moderate = 0.75, poor = 1 | 0 = 6.3  0.25 = 18.5  0.50 = 35.9  0.75 = 28.4  1 = 10.8 | - |
| Polypharmacy | < 5 medicines = 0, ≥ 5 medicines = 1 | 1 = 15.7 | - |
| Body mass index (BMI) deficit | BMI < 18.5 or BMI > 30 = 1, BMI ≥ 18.5 & BMI ≤ 30 = 0 | 1 = 25.7 | 1.2 |
| Bedrest | no = 0, yes = 1 | 1 = 8.9 | - |
| Dizziness | no = 0, yes = 1 | 1 = 20.7 | - |
| Fall(s) | no = 0, yes = 1 | 1 = 5.2 | - |
| Pain | Pain rating from 0-10. 0 = 0, ≥ 1 & ≤ 3 = 0.5, ≥ 4 = 1 | 0 = 26.1  0.5 = 35.2  1 = 38.7 | - |
| Tiredness | Never = 0,  sometimes = 0.5,  always/often = 1 | 0 = 43.0  0.5 = 42.0  1 = 15.0 | - |
| Vision | Excellent = 0, very good = 0.25, good = 0.50, moderate = 0.75, poor = 1 | 0 = 10.4  0.25 = 38.0  0.50 = 35.1  0.75 = 13.4  1 = 3.1 | 0.5 |
| Hearing | Excellent = 0, very good = 0.25, good = 0.50, moderate = 0.75, poor = 1 | 0 = 12.7  0.25 = 36.2  0.50 = 30.8  0.75 = 16.9  1 = 3.3 | 0.2 |
| Attention | 10 words immediate recall test.  ≥ 5 = 0, < 5 = 1 | 1 = 20.0 | - |
| Memory | 10 word delayed recall test.  ≥ 4 = 0, < 4 = 1 | 1 = 32.6 | - |
| Physical inactivity | Moderate physical activity: “Every day/almost every day” and “multiple times a week” = 0, “once per week” & “less often” = 1 | 1 = 21.4 | - |
| Doctor told you had: Heart problem (myocardial infarction, coronary thrombosis, other problem including congestive heart failure) | no = 0, yes = 1 | 1 = 15.3 | - |
| Doctor told you had: High blood pressure or hypertension | no = 0, yes = 1 | 1 = 48.6 | - |
| Doctor told you had: Stroke or cerebral vascular disease | no = 0, yes = 1 | 1 = 4.7 | - |
| Doctor told you had: Diabetes or high blood sugar | no = 0, yes = 1 | 1 = 19.5 | - |
| Doctor told you had: Chronic lung disease such as chronic bronchitis or emphysema | no = 0, yes = 1 | 1 = 9.9 | - |
| Doctor told you had: Cancer or malignant tumour, including leukaemia or lymphoma | no = 0, yes = 1 | 1 = 5.6 | - |
| Doctor told you had: Arthritis, including osteoarthritis, or rheumatism | no = 0, yes = 1 | 1 = 27.0 | - |
| Doctor told you had: Chronic renal disease | no = 0, yes = 1 | 1 = 2.8 | - |
| Doctor told you had: Alzheimer's disease, dementia or any other serious memory impairment | no = 0, yes = 1 | 1 = 3.1 | - |
| Lonely | never/rarely = 0  sometimes = 0.5  often/always = 1 | 0 = 76.8  0.5 = 17.6  1 = 5.6 | - |
| Difficulty concentrating | never/rarely = 0  sometimes = 0.5  often/always = 1 | 0 = 71.4  0.5 = 25.8  1 = 2.8 | - |
| Depressed | never/rarely = 0  sometimes = 0.5  often/always = 1 | 0 = 69.0  0.5 = 26.1  1 = 4.9 | - |
| Everything takes effort | never/rarely = 0  sometimes = 0.5  often/always = 1 | 0 = 66.9  0.5 = 23.9  1 = 9.2 | - |
| Sleep problems | never/rarely = 0  sometimes = 0.5  often/always = 1 | 0 = 52.7  0.5 = 35.3  1 = 12.0 | 0.2 |
| Could not get going | never/rarely = 0  sometimes = 0.5  often/always = 1 | 0 = 65.7  0.5 = 27.7  1 = 6.6 | - |
| Sad | never/rarely = 0  sometimes = 0.5  often/always = 1 | 0 = 72.3  0.5 = 23.0  1 = 4.7 | - |
| Poor appetite | never/rarely = 0  sometimes = 0.5  often/always = 1 | 0 = 89.0  0.5 = 8.0  1 = 3.1 | - |
| Weak grip strength | Grip strength (GS) = maximum grip strength in kg over four trials (2 left, 2 right).  Men:  GS ≤ 29 & BMI ≤ 24 = 1  GS ≤ 30 & BMI >24 & BMI ≤ 28 = 1  GS ≤ 32 & BMI > 28 = 1  Women:  GS ≤ 17 & BMI ≤ 23 = 1  GS ≤ 17.3 & BMI >23 & BMI ≤ 26 = 1  GS ≤ 18 & BMI > 26 & BMI ≤ 29 = 1  GS ≤ 21 & BMI > 29 = 1  Participants who cannot perform the test = 1 | 1 = 31.0 | 1.4 |
| Slow gait speed | Gait speed in seconds = maximum of two trials over 2.5 meters. Lowest 20 % = 1- Participants who cannot perform the test = 1 | 1 = 18.4 | 1.6 |
| Slow chair rises | Time in seconds for 5 chair rises. Age ≤ 79 & time > 14 = 1, age ≥ 80 & time > 16 = 1. Participants who cannot perform the test = 1 | 1 = 34.4 | 1.2 |

Supplementary Table 4: Generalized linear mixed model comparison

|  | WAIC | LOO |
| --- | --- | --- |
| Poisson | 5,735 | 5,777 |
| Negative binomial | 5,737 | 5,774 |
| Beta-binomial | 5,662 | 5,677 |
| Zero-inflated Poisson | 5,714 | 5,762 |
| Zero-inflated negative binomial | 5,724 | 5,774 |
| Zero-inflated beta-binomial | 5,647 | 5,666 |

Model weights indicated that >99% of the weight goes to the zero-inflated beta-binomial model, i.e., it represents by far the best model under both WAIC and LOO. WAIC = Watanabe Akaike Information Criterion, LOO = leave one-out cross-validation.

Supplementary Figure 2: Illustration of disability fluctuations as observation-level residuals


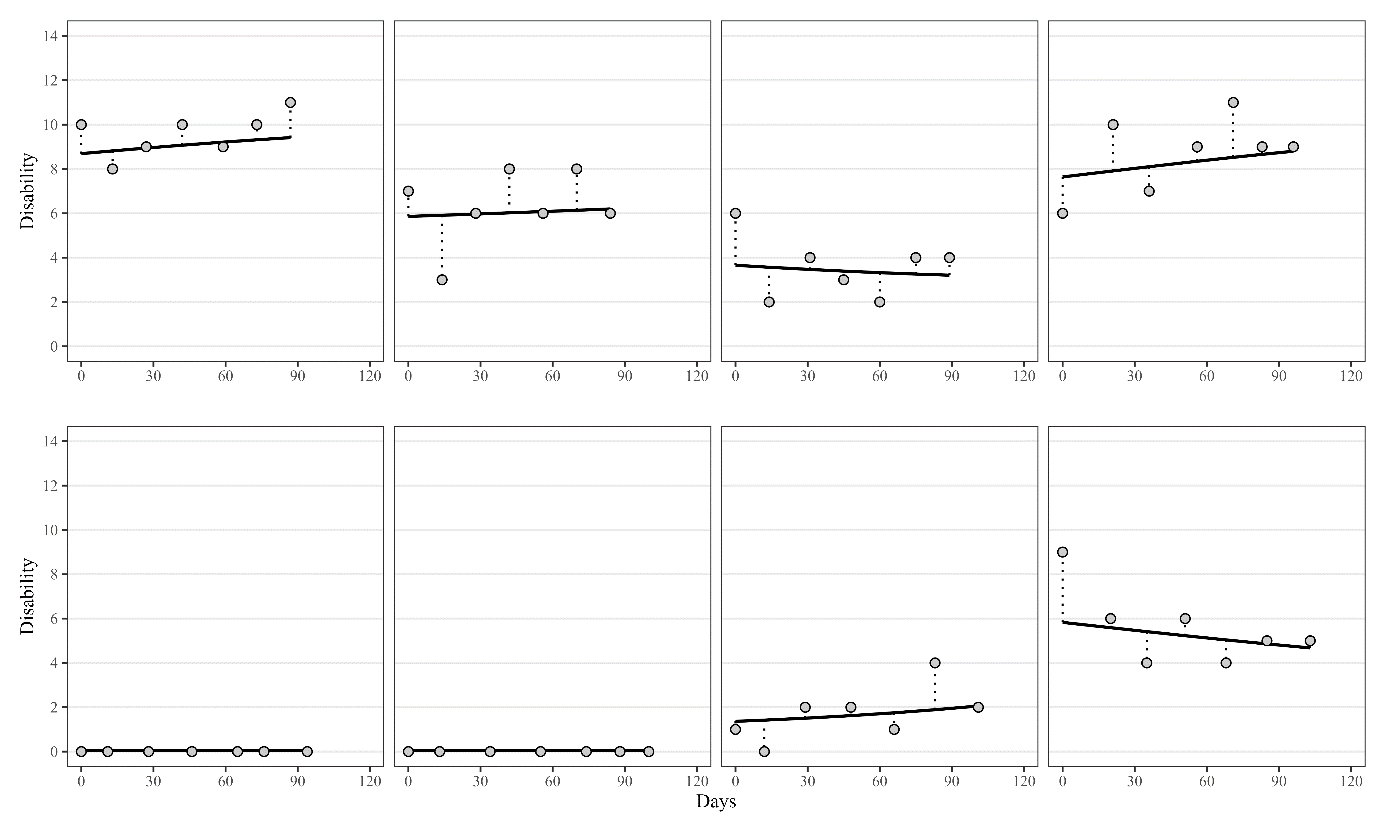


Figure shows repeated disability measurements (points) from 8 randomly selected participants (facets). The first row shows four participants who were frail at baseline (physical frailty) and the second row shows four robust participants. The solid line depicts the estimated individual disability trajectory and the dotted line shows the difference between the observed (points) and estimated value (solid line), i.e., the observation-level residuals, that is, short-term disability fluctuations.

Supplementary Methods 3: Statistical Analysis

In this study, disability was measured repeatedly as the number of activities participants reported having difficulty with during the last two weeks. A majority of study participants reported no limitations at each time point, and from those who reported difficulty with any of the 14 activities, most reported only few. The outcome disability can be characterised as strictly positive (no negative values possible), discrete (only full numbers), and highly right-skewed (skew=2.3) count variable with a fixed upper bound (=14). Therefore, linear mixed regression models that assume a normal distribution of the response variable (conditional on the random effects) would not be appropriate. Instead we considered three types of suitable generalised mixed regression models that invoke statistical distributions that start at zero and take on only whole numbers, and which are therefore often used for count data^44–47^. Specifically, we compared mixed Poisson, negative binomial and beta-binomial regression models, as well as their zero-inflated extensions, given the substantial amount of observations where participants reported no difficulty in any activity (Supplementary Figure 3). In short, the Poisson distribution models the probability of the number of events occurring at a constant rate, i.e., it uses a single parameter for both mean and variance. If the variance is considerably larger than the mean (overdispersion), this can lead to underestimated standard errors. The less restrictive two-parameter negative binomial distribution can accommodate overdispersed Poisson processes by including a gamma-distributed rate parameter. The beta-binomial distribution models the mean probability of repeated beta-distributed trial successes, which explicitly allows to incorporate an upper bound for the outcome, and also accommodates overdispersion. To accommodate excess zero values, zero-inflation extensions of all three aforementioned model types were also considered, which represent mixtures of two processes (not having any difficulty vs. having difficulty addressed by a logistic regression model, and if there are difficulties, then the number of difficulties is modelled with the aforementioned Poisson, negative binomial or beta-binomial distributions). Comparison of model fit was done based on an empty model without predictors (except for intercept and time) using the Watanabe Akaike Information Criterion (WAIC) and leave-one-out cross-validation (LOO)^48^.

In the following, we describe the two-step statistical procedure at the core of this analysis. The first set of generalized models took the following form (example below refers to a Poisson model and FP):

*µ_ij_* = exp[*β*_10_ + *β*_20_Days*_i_* + *β*_11_FP_i_ + *β*_12_Age*_i_* + *β*_13_Female*_i_ +*

*β*_14_Edu_Mid*_i_ + β*_15_Edu_High*_i_ + β*_16_Alone*_i_ + u*_1_*_i_ + u*_2_*_i_*]

where the expected number of activity limitations (*µ*) for the *i^th^* person (i = 1, ..., N) at time points *t_ij_* (j = 1, ..., *n_i_*) is estimated as the exponentiated sum of the linear predictors which include the overall intercept (*β*_10_) and slope (*β*_20_), the fixed effect of the main predictor frailty (*β*_11_) adjusted for socio-demographics (*β*_12_-*β*_16_) as well as individual-level deviations from the overall disability severity (*u*_1_*_i_*, i.e., the average disability severity of a person per burst) and slope (*u*_2_*_i_*), which take into account the correlations due to repeated observations.

In the next step, we extracted from these first-step models the observation-level residual error terms, which represent the vertical deviations from individual-specific (random intercept and slope) disability trajectories within each measurement burst, i.e., short-term disability fluctuations (Supplementary Figure 1). The extracted absolutized continuous observation-level residuals had a right-skewed distribution and were modelled as new outcome in the second step with lognormal mixed regression models as:

σ_ij_ = exp[*β*_10_ + *β*_11_FP_i_ + *β*_12_Disability*_i_* + *β*_13_Age*_i_ + β*_14_Female*_i_ +*

*β*_15_Edu:Mid*_i_ + β*_16_Edu:High*_i_ + β*_17_Alone*_i_ +u*_1_*_i_*]

where disability fluctuations (σ*)* of the *i^th^* participant (i = 1, …, N) at time points *t_ij_* (j = 1, ..., *n_i_*) are estimated as the exponentiated sum of the linear predictors which included an overall intercept (*β_10_*) and the individual deviations therefrom (*u*_1_*_i_*), frailty status (*β_11_*), the estimated average disability severity of each participant (*β*_12_) as characterised by the random intercept term from the first step model (*u*_1_*_i_*) and the same set of sociodemographic variables as above (*β*_13_-*β*_17_).

All models were estimated using R (v4.3.1). The generalized mixed regression models were estimated under a Bayesian framework using R-package brms (v2.20)^49^, an interface for the Stan programming language (v2.26). The posterior distribution was sampled using weakly informative priors and Hamiltonian Monte Carlo with three chains and 4,000 postwarm-up samples per chain. All models passed standard diagnostic criteria, i.e., $\hat{r}\leq1.01$ and bulk and tail effective sample sizes >500. Details of data preparation and all statistical analyses are documented in the R-Markdown code-file available online: https://osf.io/au5vh/.

Supplementary Table 5: Predictors of disability severity (µ) and fluctuations (σ) in the first burst

|  | Frailty phenotype | | Frailty Index | |
| --- | --- | --- | --- | --- |
|  | Disability | | Disability | |
|  | Severity | Fluctuations | Severity | Fluctuations |
|  | µ (95%-CI) | σ (95%-CI) | µ (95%-CI) | σ (95%-CI) |
| FIXED EFFECTS |  |  |  |  |
| Intercept | -11.94 (-15.29, -8.68) | -5.00 (-5.75, -4.22) | -10.61 (-13.61, -7.65) | -4.67 (-5.40, -3.92) |
| Time (days) | -0.00 (-0.00, 0.00) | - | -0.00 (-0.00, 0.00) | - |
| Age | 1.10 (1.05, 1.14) | 1.04 (1.03, 1.05) | 1.07 (1.03, 1.11) | 1.03 (1.02, 1.04) |
| Female | 2.19 (1.30, 3.50) | 1.57 (1.40, 1.76) | 2.06 (1.27, 3.18) | 1.58 (1.41, 1.77) |
| Medium education | 0.89 (0.48, 1.51) | 0.84 (0.73, 0.97) | 1.46 (0.82, 2.40) | 1.13 (0.98, 1.29) |
| High education | 0.74 (0.35, 1.36) | 0.83 (0.70, 0.97) | 1.57 (0.78, 2.82) | 1.24 (1.05, 1.45) |
| Living alone | 1.84 (1.09, 2.90) | 1.32 (1.18, 1.47) | 1.43 (0.90, 2.17) | 1.20 (1.07, 1.34) |
| Disability severity | - | 1.65 (1.60, 1.70) | - | 1.74 (1.68, 1.80) |
| Frailty | 17.76 (8.61, 32.76) | 2.48 (2.09, 2.92) | 15.31 (9.43, 22.95) | 2.84 (2.53, 3.19) |
|  |  |  |  |  |
| RANDOM EFFECTS |  |  |  |  |
| Intercept (SD) | 1.96 (1.73, 2.21) | 0.46 (0.42, 0.50) | 1.70 (1.49, 1.92) | 0.44 (0.40, 0.49) |
| Time (days) (SD) | 0.01 (0.00, 0.01) | - | 0.01 (0.00, 0.01) | - |
| Corr. Intercept*Time | 0.24 (-0.17, 0.67) | - | 0.46 (0.12, 0.79) | - |
|  |  |  |  |  |
| MODEL FIT |  |  |  |  |
| R² | 0.38 | 0.34 | 0.24 | 0.38 |
| WAIC | 5,687 | -2,057 | 5,688 | -2,039 |

Results from mixed zero-inflated beta-binomial (disability severity) and mixed lognormal (disability fluctuations) regression models based on 425 participants and 2,891 observations. Coefficients are exponentiated (except for intercept and time) point estimates (mean posterior distribution). Effective sample size > 500 for all parameters, and R-hat=1.0. 95%-CI = 95% credible intervals, µ = average disability severity, σ = short-term disability fluctuations, Corr. = correlation, R² = Bayesian R-squared based on fixed effects, WAIC = Watanabe Akaike Information Criterion.

Supplementary Table 6: Predictors of disability severity (µ) and fluctuations (σ) in the second burst

|  | Physical frailty (FP) | | Frailty Index (FI) | |
| --- | --- | --- | --- | --- |
|  | Disability | | Disability | |
|  | Severity | Fluctuations | Severity | Fluctuations |
|  | µ (95%-CI) | σ (95%-CI) | µ (95%-CI) | σ (95%-CI) |
| FIXED EFFECTS |  |  |  |  |
| Intercept | -13.76 (-17.99, -9.73) | -6.03 (-7.08, -4.96) | -12.90 (-16.92, -8.89) | -5.76 (-6.81, -4.73) |
| Time (days) | 0.00 (0.00, 0.01) | - | 0.00 (-0.00, 0.01) | - |
| Age | 1.13 (1.07, 1.19) | 1.06 (1.04, 1.07) | 1.10 (1.05, 1.16) | 1.05 (1.04, 1.06) |
| Female | 1.81 (1.01, 3.33) | 1.15 (0.99, 1.35) | 1.83 (0.98, 3.17) | 1.16 (0.99, 1.35) |
| Medium education | 0.90 (0.41, 1.74) | 0.87 (0.71, 1.05) | 1.46 (0.67, 2.83) | 1.05 (0.85, 1.27) |
| High education | 0.70 (0.78, 1.47) | 0.74 (0.59, 0.93) | 1.42 (0.56, 3.01) | 0.99 (0.78, 1.23) |
| Living alone | 1.48 (0.81, 2.51) | 1.13 (0.96, 1.31) | 1.26 (0.68, 2.15) | 1.08 (0.92, 1.26) |
| Disability severity | - | 1.47 (1.43, 1.52) | - | 1.51 (1.46, 1.57) |
| Frailty | 16.65 (6.47, 36.70) | 2.01 (1.55, 2.58) | 11.39 (5.92, 19.78) | 2.38 (2.02, 2.80) |
|  |  |  |  |  |
| RANDOM EFFECTS |  |  |  |  |
| Intercept (SD) | 2.45 (0.214, 2.78) | 0.61 (0.55, 0.66) | 2.26 (1.98, 2.57) | 0.62 (0.56, 0.67) |
| Time (days) (SD) | 0.01 (0.00, 0.01) | - | 0.01 (0.00, 0.01) | - |
| Corr. Intercept*Time | -0.40 (-0.70, -0.04) | - | -0.15 (-0.51, 0.30) | - |
|  |  |  |  |  |
| MODEL FIT |  |  |  |  |
| R² | 0.29 | 0.20 | 0.20 | 0.20 |
| WAIC | 4,356 | -1,859 | 4,354 | -2.332 |

Results from mixed beta-binomial (level) and mixed lognormal (fluctuations) regression models based on 375 participants and 2,192 observations. Coefficients are exponentiated (except for the intercept term) point estimates based on the mean posterior distribution. Effective sample size > 500 for all parameters, and R-hat=1.0. 95%-CI = 95% credible intervals, µ = average disability severity, σ = short-term disability fluctuations, Corr. = correlation, R² = Bayesian R-squared based on fixed effects, WAIC = Watanabe Akaike Information Criterion.

Supplementary Figure 3: Distribution of the number of difficulties in activities


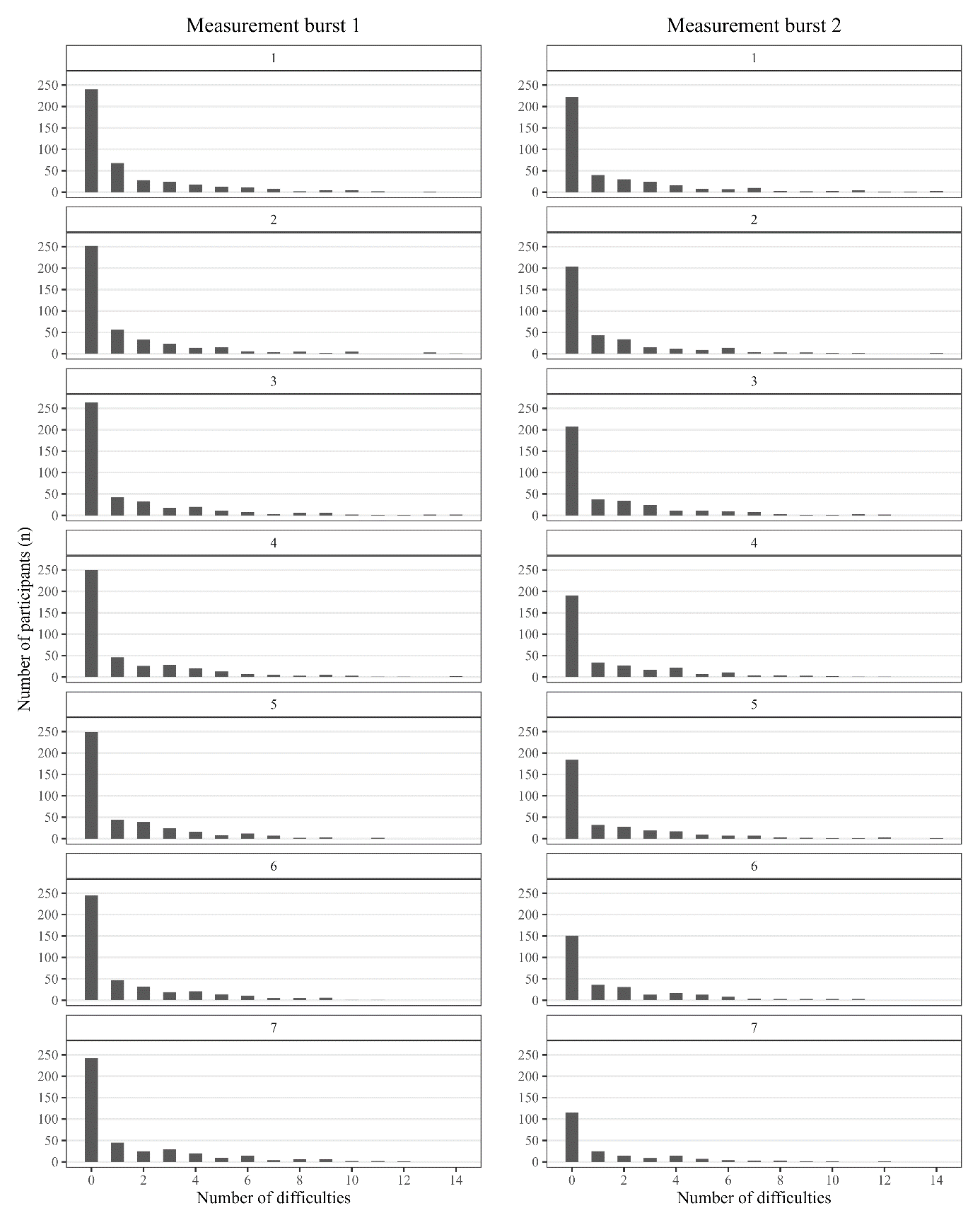


Supplementary References

42. Revelle W, Condon DM. Reliability from α to ω: A tutorial. *Psychological Assessment*. 2019;31(12):1395-1411. doi:10.1037/pas0000754

43. Rosseel Y. lavaan: An R Package for Structural Equation Modeling. *Journal of Statistical Software*. 2012;48:1-36. doi:10.18637/jss.v048.i02

44. Agresti A. *Categorical Data Analysis*. 1st ed. Wiley; 2002. doi:10.1002/0471249688

45. Molenberghs G, Verbeke G, Demétrio CGB. An extended random-effects approach to modeling repeated, overdispersed count data. *Lifetime Data Anal*. 2007;13(4):513-531. doi:10.1007/s10985-007-9064-y

46. Du J, Park YT, Theera-Ampornpunt N, McCullough JS, Speedie SM. The use of count data models in biomedical informatics evaluation research. *J Am Med Inform Assoc*. 2012;19(1):39-44. doi:10.1136/amiajnl-2011-000256

47. Green JA. Too many zeros and/or highly skewed? A tutorial on modelling health behaviour as count data with Poisson and negative binomial regression. *Health Psychol Behav Med*. 9(1):436-455. doi:10.1080/21642850.2021.1920416

48. Gelman A, Hwang J, Vehtari A. Understanding predictive information criteria for Bayesian models. *Stat Comput*. 2014;24(6):997-1016. doi:10.1007/s11222-013-9416-2

49. Bürkner PC. brms: An R Package for Bayesian Multilevel Models Using Stan. *J Stat Software*. 2017;80:1-28. doi:10.18637/jss.v080.i01
